# Tumor in Mammary Gland

**Published:** 1872-11

**Authors:** 


### Tumor in Mammary Gland.

An apparently healthy married lady of fifty-three years appeared at this clinic on the 15th of last month, on acconnt of a painful tumor of the right breast, which she had first noticed only two months before, since which time it had increased considerably in size, and occasioned her much more anxiety. She could not bear the pressure of a tight dress upon it, as this increased the pain.

The lecturer proceeded to examine the patient. We find a circumscribed, movable tumor, rather deep-seated, and external to the nipple. It is nodulated, but not uniformly hard; in places it appears softer, as if it contained small cysts with fluid contents. Although appearing of considerable bulk, yet when the surrounding adipose and cellular tissue are dissected off, it will be of rather small size. The breast is freely movable; soft, except in the affected spot, and the nipple is natural and not retracted; the skin being healthy and not adherent or attached to the tumor. The pain is sharp and neuralgic in character, and the patient thinks it is worse in wet weather. This tumor strongly resembles a gland undergoing fibroid degeneration, but I cannot state more positively in regard to it, until I can make a section of it before you, on a plate. There are no enlarged glands in the neighborhood of the tumor, or in the axilla.

This is not cancer. You might expect to find a scirrhus of the breast in a woman of this age, as it generally comes on soon after the decline of the menses. In scirrhus the tumor adheres to the surrounding structure, so that by the time it attains this size it is not freely movable, as this one is. The skin also would be discolored and brawn-like, and the nipple retracted early in the disease, forming a prominent diagnostic sympton. The glands of the axilla in scirrhous disease become involved, and the morbid structure soon breaks down and forms one or more irregular, foul-looking ulcers, with hard depressed edges, discharging a sanious, offensive, acrid fluid. The affection before us does not resemble encepheloid disease, which is comparatively rare in the mammary gland; nor is it like epithelioma, colloid, or melanosis, which also are extremely uncommon in this situation. Adenoma and fibroma frequently occur here, but are mostly met with in young girls, shortly after puberty, in whom they generally co-exist, and may be connected with dysmenorrhæal troubles and a nervous temperament. It also occurs, occasionally, soon after marriage and during the first pregnancy. We might use in its treatment the cicuta plaster or the opium plaster, or palliatives, or sorbefacient applications; but the only effectual remedy is the knife.

Chloroform was administered and the tumor removed; part of it was hard, dense, and resisting to the knife, white in color, and resembled a degenerated lymphatic gland. Throughout the greater part of its bulk it contained a number of cysts of varying sizes, in which was a dark-colored, jelly-like fluid.

September 21, the patient was again presented. A slight erysipelas had supervened upon the operation, but had yielded promptly to remedies. This erysipelas may have been produced by a number of causes, which we will not recount, as it could at best be nothing but conjecture. A tendency to erysipelas seems to exist in some persons, particularly those of a nervous, irritable temperament; it is also seen in intemperates. This affection prevails more at some seasons of the year than at others, and is sometimes endemic in certain localities. S)me peculiarty of the atmosphere, which sometimes exists in hospitals, seems to exert a powerful effect, both in predisposing patients to the disease and producing an attack, Simple erysipelas exhibts itself as a bright red discoloration of the skin, which has a marked tendency to spread. In its treatment we may use equal parts of tincture of iodine and alcohol, which may be painted on with a brush twice or thrice daily; or a cloth may be kept on the part wet with a solution of muriate of ammonia, acetate of lead, and opium, or even cold water. In the phlegmonous variety, and in that form supervening upon a wound or ulcer, which involve structure beneath the epidermis, the treatment by vesication is particularly beneficial.

The tumor was carefully examined by Dr. R. M. Bertolet, who made the following report:

"The mammary tumor, which is made up of a number of small cysts filled with a dark, straw-colored, gelatinous fluid, is a *cysto-sarcoma mucosum.* The microscopial appearances are briefly as follows:

"The dilated lacteal ducts and the attenuated and distorted glandular acini form the round cystic spaces. These are filled with a fluid of a semi-solid consistence, resembling the jelly of Wharton, and composed of a meshwork of anastomosing stellate cells, with a hyaline intercellular substance which is rendered cloudy upon the addition of acetic acid. The remains of theoriginal lining of the cylinder epithelium are now and then seen upon the walls of the cysts. From the walls of the glands are seen springing many papila-like proliferations, also generally deprived of their epithelial covering.

"Throughout the growth are imbedded numerous sarcomatous cells of the small-celled variety."

We can assure this patient that the disease, in all probability, will not return; as circumscribed sarcoma (including the fibroid and cystic varieties) is the least malignant of all its forms.—*Med. Times.*

